# RTVP-1 promotes mesenchymal transformation of glioma via a STAT-3/IL-6-dependent positive feedback loop

**DOI:** 10.18632/oncotarget.4205

**Published:** 2015-07-18

**Authors:** Nis David Giladi, Amotz Ziv-Av, Hae Kyung Lee, Susan Finniss, Simona Cazacu, Cunli Xiang, Hiba Waldman Ben-Asher, Ana deCarvalho, Tom Mikkelsen, Laila Poisson, Chaya Brodie

**Affiliations:** ^1^ Everard and Mina Goodman Faculty of Life Sciences, Bar-Ilan University, Ramat-Gan, Israel; ^2^ Department of Neurosurgery, Davidson Laboratory of Cell Signaling and Tumorigenesis, Hermelin Brain Tumor Center, Henry Ford Hospital, Detroit, MI, USA; ^3^ Department of Public Health Sciences, Henry Ford Hospital, Detroit, MI, USA

**Keywords:** glioblastoma, glioma stem cells, RTVP-1, IL-6, mesenchymal transformation

## Abstract

Glioblastomas (GBMs), the most aggressive primary brain tumors, exhibit increased invasiveness and resistance to anti-tumor treatments. We explored the role of RTVP-1, a glioma-associated protein that promotes glioma cell migration, in the mesenchymal transformation of GBM. Analysis of The Cancer Genome Atlas (TCGA) demonstrated that RTVP-1 expression was higher in mesenchymal GBM and predicted tumor recurrence and poor clinical outcome. ChiP analysis revealed that the RTVP-1 promoter binds STAT3 and C/EBPβ, two master transcription factors that regulate mesenchymal transformation of GBM. In addition, IL-6 induced RTVP-1 expression in a STAT3-dependent manner. RTVP-1 increased the migration and mesenchymal transformation of glioma cells. Similarly, overexpression of RTVP-1 in human neural stem cells induced mesenchymal differentiation, whereas silencing of RTVP-1 in glioma stem cells (GSCs) decreased the mesenchymal transformation and stemness of these cells. Silencing of RTVP-1 also increased the survival of mice bearing GSC-derived xenografts. Using gene array analysis of RTVP-1 silenced glioma cells we identified IL-6 as a mediator of RTVP-1 effects on the mesenchymal transformation and migration of GSCs, therefore acting in a positive feedback loop by upregulating RTVP-1 expression via the STAT3 pathway. Collectively, these results implicate RTVP-1 as a novel prognostic marker and therapeutic target in GBM.

## INTRODUCTION

Glioblastoma (GBM) are the most common and aggressive astrocytic tumors and are characterized by increased proliferation, invasion into the surrounding normal tissue, robust angiogenesis and resistance to conventional therapies [1]. The prognosis of patients with GBM remains extremely poor and has not changed significantly during the past several years [2, 3]. GBM contain a small subpopulation of cancer stem cells (i.e., glioma stem cells [GSCs]) [4, 5] that are characterized by self-renewal ability, multi-lineage differentiation potential and the ability to generate tumors that recapitulate the parental tumors [6]. In recent years, GSCs have been implicated in treatment resistance and tumor recurrence of GBM [7].

Recently, gene expression profiling studies have identified four GBM subtypes that were classified based on their transcriptional signatures into proneural, neural, classical and mesenchymal subtypes [8–12]. These subtypes have distinct differential genetic alterations, molecular signature, cellular phenotypes and patient prognosis. [9, 13–15].

Recent studies identified the transcription factors (TFs), signal transducer and activator of transcription 3 (STAT3) and CCAAT enhancer-binding protein β (C/EBPβ) as synergistic initiators and master regulators of the mesenchymal transformation in GBM [16]. The expression of C/EBPβ and STAT3 correlates with the mesenchymal phenotype of GBM and predicts poor clinical outcome and these two TF have been demonstrated to regulate the mesenchymal transformation of glioma cells [15, 16]. The downstream mechanisms by which these TFs mediate and maintain the mesenchymal phenotype of glioma are just beginning to be identified.

Related to testis-specific, vespid and pathogenesis protein 1 (RTVP-1) was originally cloned from human GBM cell lines by two groups and was termed glioma pathogenesis-related protein - GLIPR1 [17] or RTVP-1 [18]. RTVP-1 contains a putative signal peptide, a transmembrane domain and a SCP domain, with a yet unknown function. This domain is also found in other RTVP-1 homologs including TPX-1 [19], the venom allergen antigen [20] and group 1 of the plant pathogenesis-related proteins (PR-1). We recently reported that RTVP-1 is not detected in normal brain specimens, is highly expressed in astrocytic tumors and that the expression of RTVP-1 correlates with the degree of malignancy of these tumors. Moreover, overexpression of RTVP-1 increases glioma cell proliferation, invasion and anchorage-independent growth, whereas its silencing induces apoptosis in glioma cells [21]. RTVP-1 expression is upregulated by the tumor-promoting protein kinase C (PKC) isoforms PKCα and PKCε while PKCδ exerts an opposite effect [22]. The regulation of RTVP-1 by these PKC isoforms was at least partly mediated by the TF serum response factor (SRF) which is known to be differently regulated by these PKC isoforms. In addition, RTVP-1 expression in GBM is regulated by hypermethylation [23].

RTVP-1 has been also reported to be highly expressed in invasive melanoma cells and to regulate melanoma cell invasion [24]. In addition, high expression levels of RTVP-1 and its association with tumorigenesis have been reported in Wilms' tumors [25]. In contrast, RTVP-1 functions as a tumor suppressor in prostate cancer [26].

Here, we studied the function of RTVP-1 in the mesenchymal transformation of GBM and found that it was highly expressed in the mesenchymal GBM subtype compared to other subtypes and its expression was induced by and correlated with the expression of STAT3 and C/EBPβ. Moreover, we found that RTVP-1 regulated the mesenchymal transformation of both glioma cell lines and GSCs and that some of its effects were mediated by IL-6.

## RESULTS

### RTVP-1 is highly expressed in the mesenchymal subtype of GBM and predicts poor clinical outcome

**Figure 1:**
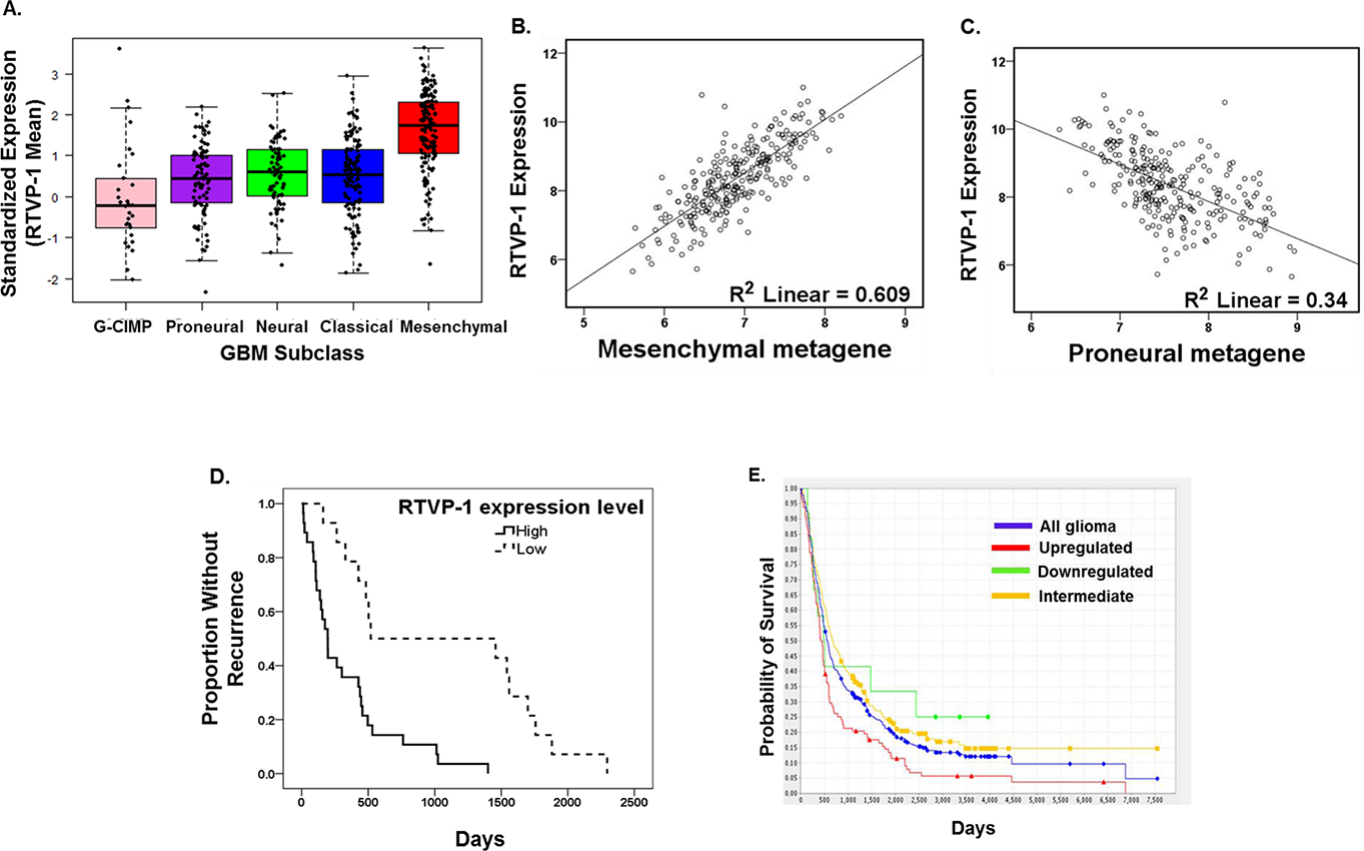
RTVP-1 is highly expressed in the mesenchymal subtype of GBM and predicts poor clinical outcome Upregulated RTVP-1 expression vs. all other samples, *P* > 0.0001. RTVP-1 gene expression in the five GBM subtypes of 481 GBM specimens was analyzed using the TCGA data portal as described in the methods **A.** Scatter plot of RTVP-1 expression versus the mesenchymal **B.** and the proneural **C.** metagene scores in TCGA GBM dataset of 259 patients. Details regarding the calculation of the metagene score and its definition are described in the method section. Kaplan-Meier curves comparing patients with GBM expressing high vs. low levels of RTVP-1 were determined using TCGA dataset **D.** The tumor samples were partitioned into two groups of equal size depending on their levels of RTVP-1. Shown are the Kaplan-Meier curves for the corresponding samples with entries in the “Days to Tumor Recurrence” field. *P*-values for change in survival are assessed by a log-rank test. *P* < 0.00014. Kaplan-Meier survival plot for 343 GBM patients with differential RTVP-1 gene expression was analyzed using REMBRANDT **E.**

We previously reported that RTVP-1 is expressed in astrocytic tumors in a grade-dependent manner but is almost undetectable in normal brain [21]. Moreover, RTVP-1 regulated the proliferation and invasion of glioma cells [22]. In this study, we further examined the role of RTVP-1 in glioma cell migration and in the mesenchymal transformation of these cells. We first examined whether RTVP-1 expression was associated with the mesenchymal subtype of GBM, which is known to be associated with increased degree of infiltration and predicts poor clinical outcome. Using The Cancer Genome Atlas (TCGA data portal) [27], we analyzed the relative expression of RTVP-1 in the different subtypes of GBM. We found that the mean expression of RTVP-1 was significantly higher in the mesenchymal subtype (*P* < 0.0001) compared to the proneural, GCIMP, neural and the classical GBM subtypes (Fig. 1A), whereas its expression was significantly lower in the GCIMP subtype compared with the other GBM subtypes (Fig. 1A, Suppl. Table S1). Moreover, as presented in Fig. 1B and 1C, RTVP-1 expression in GBM was positively correlated with the mesenchymal metagene score (Pearson correlation 0.78, *P* < 0.0001) and negatively correlated with the proneural metagene score (Pearson correlation −0.583, *P* < 0.0001); both were generated from the recently reported mesenchymal and proneural genes lists [10]. These analyses indicate that RTVP-1 is preferentially expressed in the mesenchymal subtype of GBM and may have a role in the proneural-to-mesenchymal transformation of these tumors.

Using the TCGA data [27], we also found that patients with GBM expressing low levels of RTVP-1 have a significantly prolonged disease-free survival compared to patients with tumors expressing high levels of this protein (1062 days vs. 333 days, *P* = 0.00014) (Fig. 1D). Interestingly, low expression of RTVP-1 in GBM tumors is a more significant predictive factor of prolonged disease-free survival than the absence of mesenchymal gene expression signature (Fig. S1). We also used the REMBRANDT (Repository of Molecular Brain Neoplasia Data) [28] data portal and found that high expression of RTVP-1 was significantly associated with worse clinical outcome compared with tumors expressing either intermediate or low levels of RTVP-1 (Fig. 1E).

### The transcription factors C/EBPβ and STAT3 bind to and regulate RTVP-1 expression

**Figure 2:**
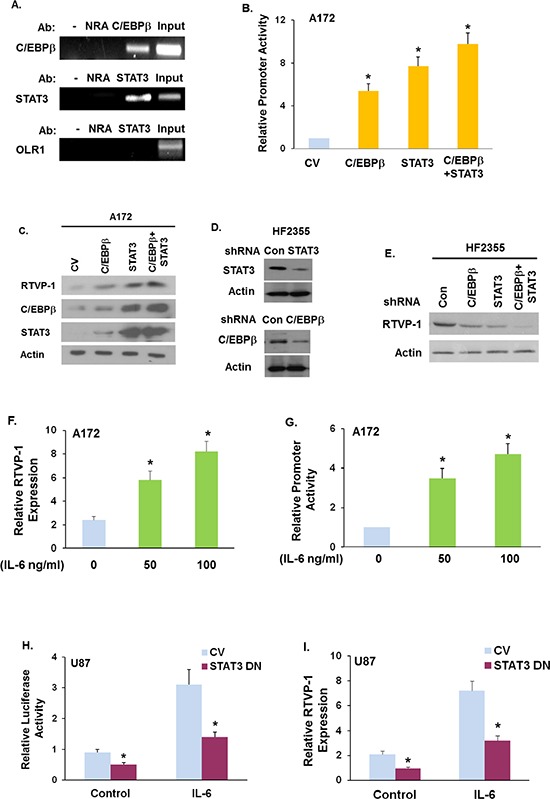
The TFs C/EBPβ and STAT3 and IL-6 regulate RTVP-1 expression Chromatin immunoprecipitation (ChIP) assay was performed according to the manufacturer's instruction using anti-STAT3 or anti-C/EBPβ antibodies **A.** Anti-GST antibody was used as a control non-relevant antibody (NRA). The specific region in the RTVP-1 promoter was amplified by PCR using specific primers. Total chromatin before immunoprecipitation was used as positive control for the PCR (INPUT) and the OLR1 gene was used as a negative control (A) A172 cells were co-transfected with C/EBPβ, STAT3 or C/EBPβ + STAT3 plasmids and with the RTVP-1 promoter cloned into luciferase vector for 48 h. Dual-luciferase reporter assays were conducted and the results were normalized to Renilla luciferase activity as a control for transfection efficiency and cell number **B.** C/EBPβ, STAT3, STAT3+C/EBPβ or a control vector were overexpressed in A172 cells **C.** or silenced in the HF2355 GSCs **D.** and the expression of RTVP-1 was measured by Western blot analysis C, **E.** U87 cells were treated with IL-6 (50 or 100 ng/ml) and the expression of RTVP-1 was determined by qRT-PCR **F.** A172 cells transfected with the RTVP-1 promoter in luciferase plasmid were treated with IL-6 **G.** U87 cells were overexpressed with a STAT3 Y705F mutant followed by treatment with IL-6 (50 ng/ml, **H.**). Dual luciferase reporter assays were performed and the results were normalized to Renilla luciferase activity as a control for transfection efficiency G, H. The expression of RTVP-1 mRNA in these samples was determined by qRT PCR **I.** The results are representative of three different experiments that gave similar results A, C, D, E, F, I or are the means ± SE of three different experiments B, G, H.*P < 0.01.

We next examined whether RTVP-1 is regulated by C/EBPβ and STAT3, the two transcription factors that were recently reported as master regulators of the mesenchymal transformation of glioma [16]. Analyzing RTVP-1 promoter for transcriptional regulatory elements using the MatInspector software revealed several different putative binding sites for C/EBPβ and STAT3 (Fig. S2). Using chromatin immunoprecipitation (ChIP) assay, we further validated that the RTVP-1 promoter binds both C/EBPβ and STAT3 in the U87 glioma cells (Fig. 2A).

We next examined the effects of C/EBPβ, STAT3 and C/EBPβ + STAT3 overexpression on the promoter activity of RTVP-1. Cloning and characterization of the RTVP-1 promoter was recently reported [21]. For these experiments we co-overexpressed the above TFs alone and in combination with a RTVP-1 promoter fragment that was cloned into a luciferase-based vector as described previously [22]. Overexpression of C/EBPβ, STAT3 or C/EBPβ + STAT3 in A172 glioma cells (that express low levels of RTVP-1), increased the promoter activity of RTVP-1 as measured by luciferase assay (Fig. 2B) and the expression of RTVP-1 (Fig. 2C), whereas, silencing of C/EBPβ, STAT3 or C/EBPβ + STAT3 in the primary GSCs HF2355 (Fig. 2D) downregulated RTVP-1 expression (Fig. 2E). Similar effects were obtained with additional shRNA constructs (data not shown).

To further analyze the regulation of RTVP-1 expression in glioma cells we employed IL-6 which phosphorylates and activates STAT3. Treatment of A172 glioma cells with IL-6 upregulated the expression of RTVP-1 in glioma cells (Fig. 2F) and the activity of the RTVP-1 promoter (Fig. 2G). To examine the role of STAT3 activation in the induction of RTVP-1 by IL-6, we employed a STAT3 dominant negative mutant in which tyrosine 705 was mutated to phenylalanine (STAT3 DN). Overexpression of the STAT3 DN in the U87 glioma cells abrogated the increased promoter activity (Fig. 2H) and the expression of RTVP-1 induced by IL-6 (Fig. 2I). Moreover, using the TCGA data set we found that RTVP-1 expression in GBM specimens was positively correlated with the expression of STAT3 (Fig. S3A), C/EBPβ (Fig. S3B) and C/EBPβ + STAT3 expression (Suppl. Fig. S3C) (Pearson correlation: 0.728, 0.349, 0.673 respectively, *P* < 0.0001 for all).

### RTVP-1 induces and is required for the maintenance of the mesenchymal phenotype in glioma cells

**Figure 3:**
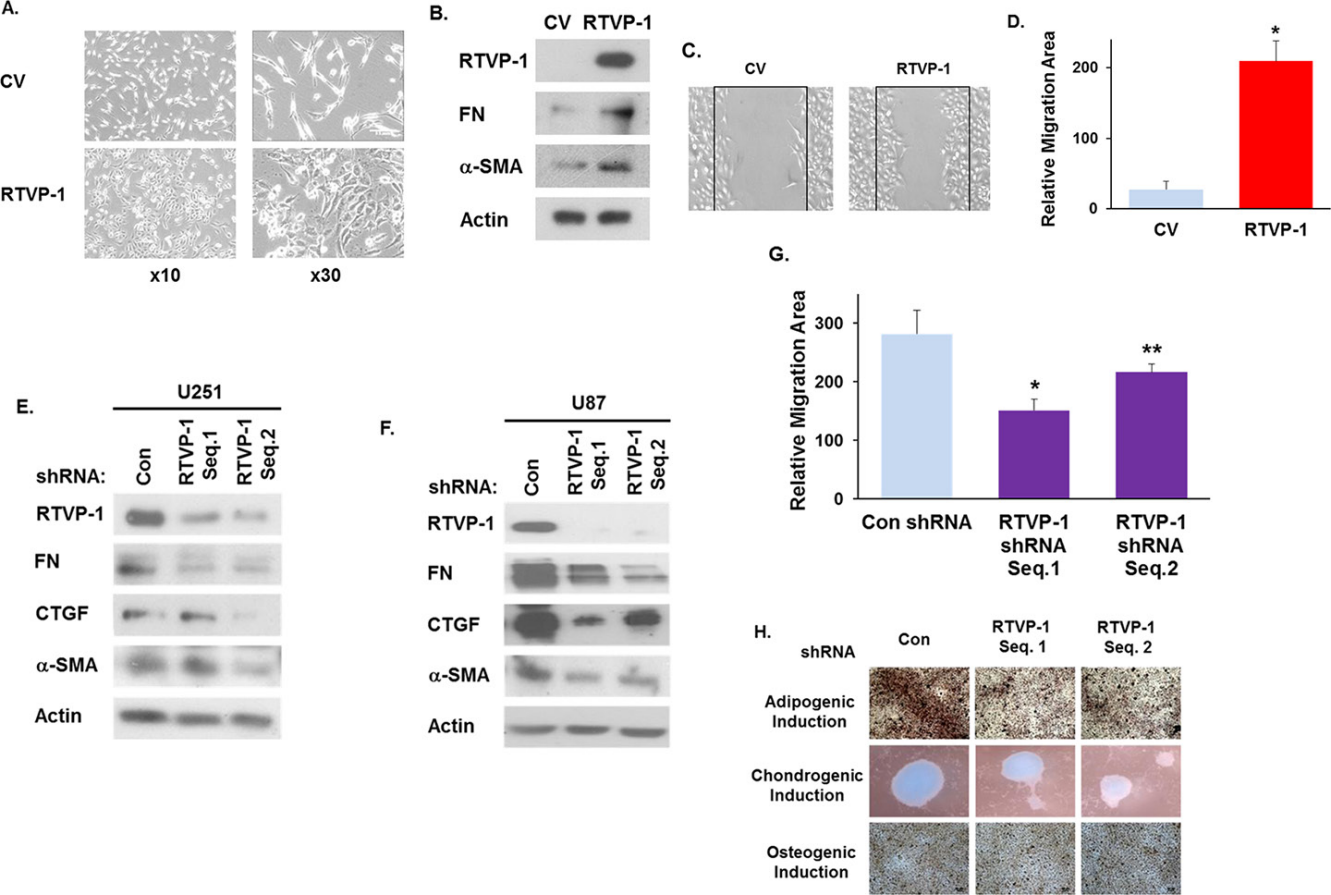
RTVP-1 induces and is required for maintaining the mesenchymal phenotype of glioma cells A172 cells were transduced with a lentivirus vector expressing RTVP-1. The cells were analyzed for cell morphology after 72 h **A.** and the expression of the mesenchymal marker proteins FN and α-SMA was determined using Western blot analysis **B.** Cell migration was analyzed 16 h later by scratch assay and images were acquired using OLYMPUS IX50 microscope and 4x objective **C.** The area of cell migration was analyzed using ImageJ software **D.** *P < 0.001. U251 **E.** and U87 **F.** cells were silenced with two different RTVP shRNAs and were analyzed for the expression of FN, CTGF and α-SMA using Western blot analysis. U87 cells were silenced with two RTVP-1 shRNAs for 48 h, cell migration was determined using a scratch assay and the area of cell migration was analyzed using ImageJ software **G.** U87 cells were silenced with two different RTVP shRNAs and were cultured with induction media for three mesenchymal lineages. 21 days later, cells were stained with oil O-red, alcian blue and alizarin red S for evaluation of adipogenic, chondrogenic and osteogenic differentiations, respectively **H.** The results are representative of three different experiments that gave similar results. *P < 0.001 **P < 0.01.

After demonstrating that RTVP-1 is preferentially expressed in the mesenchymal subtype of GBM and is positively regulated by C/EBPβ and STAT3, we next examined if RTVP-1 plays a role in regulating the mesenchymal phenotype of glioma cells. Overexpression of RTVP-1 in A172 glioma cells induced a mesenchymal-like morphology (Fig. 3A), upregulated the mesenchymal markers fibronectin 1 (FN) and α-SMA (Fig. 3B) and increased cell migration of these cells (Fig. 3C–3D). Silencing of RTVP-1 in the two human glioma cell lines, U251 and U87 resulted in downregulation of the mesenchymal markers FN, α-SMA and CTGF (Fig. 3E-3F) and in the inhibition of U87 glioma cell migration (Fig. 3G). Moreover, silencing of RTVP-1 abrogated the adipogenic and chondrogenic differentiation of these cells in response to specific induction media (Fig. 3H). Taken together, these results demonstrate that RTVP-1 induces and is required for the mesenchymal phenotype of glioma cells.

### RTVP-1 promotes and maintains the mesenchymal phenotype and stemness of GSCs

**Figure 4:**
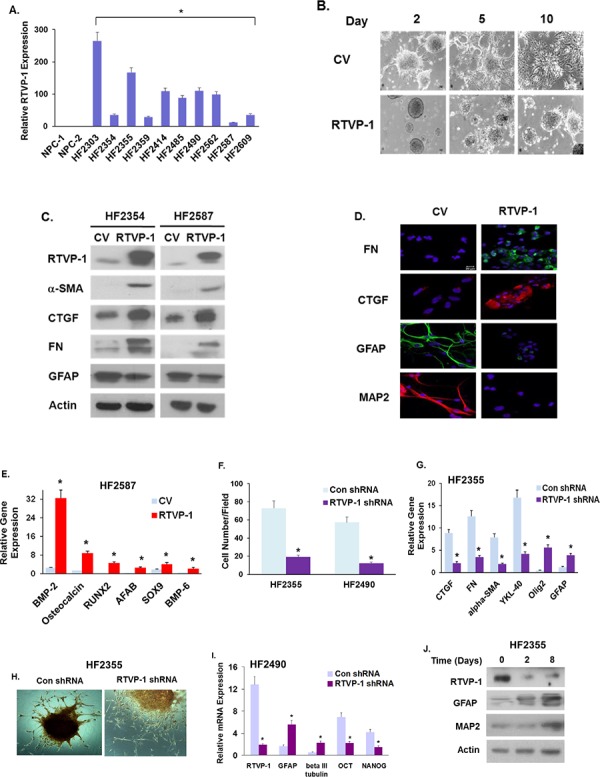
RTVP-1 induces a proneural to mesenchymal transformation of GSCs and is required for maintaining the mesenchymal phenotype of these cells The expression of RTVP-1 in ten different primary GSCs that were established from GBM specimens and in two hNSCs was analyzed using qRT-PCR **A.** GSCs were transduced with lentivirus vectors expressing RTVP-1 or a control vector, plated on poly-L-lysine and cultured under neural differentiation condition for 10 days. The morphological differentiation of the cells **B.** and the expression of different neural and mesenchymal markers were analyzed using Western blot analysis **C.** or by immunofluorescence staining **D.** GSCs overexpressing RTVP-1 were also analyzed for the expression of different mesenchymal markers using qRT-PCR **E.** GSCs were transduced with lentivirus vectors expressing RTVP-1 or control shRNAs and analyzed for cell migration using transwell migration assay **F.** The expression of various neural and mesenchymal markers was measured in the HF2355 GSCs cells using qRT- PCR **G.** The RTVP-1 silenced GSCs were cultured with adipogenic induction medium for 24 days and then stained with alizarin for the analysis of their osteogenic differentiation **H.** The results are representative of three different experiments that gave similar results. *P < 0.001. GSCs silenced for RTVP-1 were analyzed for the expression of stemness markers using qRT-PCR. *P* < 0.01 **I.** GSCs were plated on poly-L-lysine and cultured under neural differentiation condition and the expression of RTVP-1 and neural markers were analyzed using Western blot analysis **J.** The results are representative of three different experiments that gave similar results.

Since GSCs play a major role in GBM migration, therapy resistance and tumor recurrence, we examined the role of RTVP-1 also in the mesenchymal transformation of these cells. As previously reported [29], RTVP-1 was not expressed in human NSCs but was expressed to a different degree in various GSC cultures (Fig. 4A). Overexpression of RTVP-1 in the HF2587 and HF2354 GSCs (that express low levels of this protein), inhibited their neural differentiation when incubated in a differentiation medium, as indicated by their morphology (Fig. 4B), decreased the expression of the neural markers GFAP and MAP2 and upregulated the expression of the mesenchymal markers FN, α-SMA and CTGF (Fig. 4C and 4D) and genes that are associated with specific mesenchymal lineages (Fig. 4E).

To further elucidate the role of RTVP-1 in regulating the mesenchymal phenotype of GSCs, we employed lentivirus vectors expressing shRNAs targeting RTVP-1. Silencing of RTVP-1 in the HF2355 and HF2490 GSCs markedly decreased cell migration (Fig. 4F), downregulated the expression of the mesenchymal markers CTGF, FN, α-SMA and YKL-40 and upregulated the expression of the neural markers Olig2 and GFAP (Fig. 4G). Furthermore, silencing of RTVP-1 decreased the osteogenic differentiation of GSCs (Fig. 4H).

During the process of mesenchymal transformation, cancer cells acquire stem cell-like characteristics including increased self-renewal ability, migration and formation of micro metastases [30]. Similarly, the self-renewal ability is a hallmark of GSC stemness [31, 32]. We recently demonstrated that RTVP-1 promoted the self-renewal and stemness of GSCs via the activation of the CXCR4/Shh/Gli/Nanog pathway [29]. We further demonstrated now that silencing of RTVP-1 in GSCs decreased the expression of Nanog, Sox2, OCT4 and CD44 (Fig. 4I). During differentiation, GSCs lose their stemness and differentiate into the different neural lineages; a process that is characterized by the loss of stemness markers and acquisition of neural markers. We found that during GSC differentiation, the expression of endogenous RTVP-1 protein was significantly decreased along with the acquisition of the neural markers GFAP and MAP2 (Fig. 4J). Altogether, these results indicate that RTVP-1 has a functional role in the maintenance of GSCs and their stemness potential.

### RTVP-1 induces mesenchymal differentiation of human NSCs

**Figure 5:**
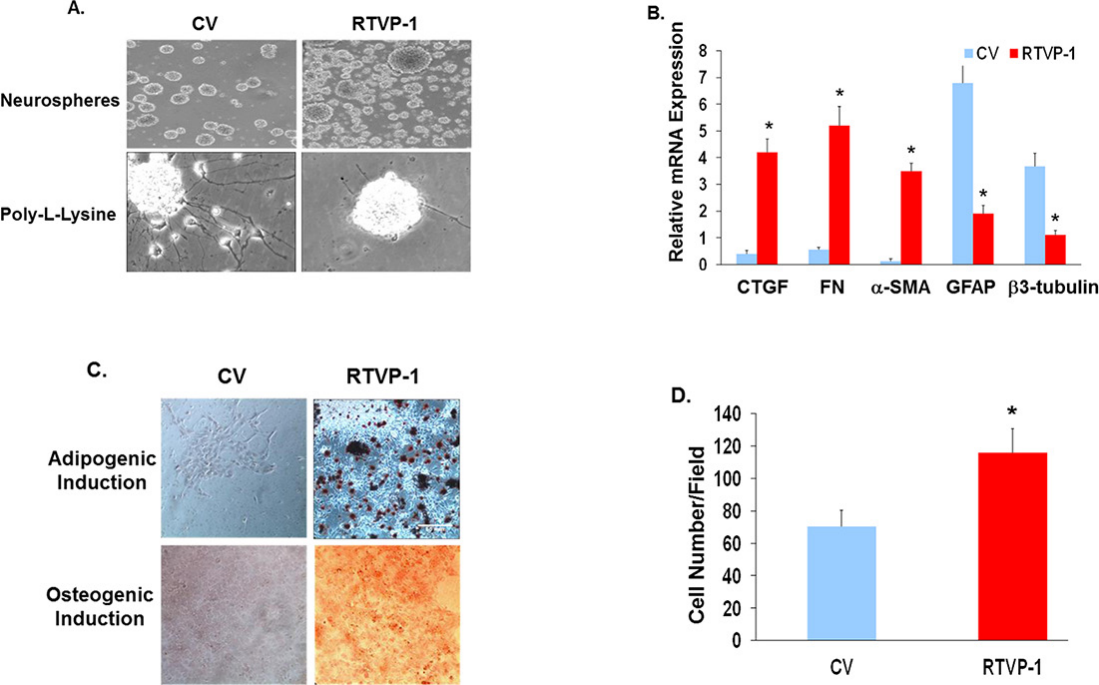
RTVP-1 induces mesenchymal differentiation of hNSCs hNSCs were transduced with lentivirus vectors expressing RTVP-1 or a control vector. The cells were maintained as spheroids or plated on poly-L-lysine and cultured in neural differentiation medium for 5 days. Cell morphology was determined using phase contrast microscopy **A.** The expression of mesenchymal and neural markers was determined using qRT-PCR **B.** The cells were also cultured in induction media for mesenchymal differentiation. Twenty-one days later, cells were stained with oil O-red and alizarin red S for the evaluation of adipogenic and osteogenic differentiation, respectively **C.** Cell migration of the RTVP-1 overexpressing cells was determined using transwell migration assay after 8 h of incubation **D.** The results are representative of three different experiments that gave similar results. *P < 0.001.

Since glioma cells and GSCs express different aberrant pathways and genetic alterations that could cooperate with RTVP-1 to induce and maintain mesenchymal transformation, we examined whether overexpression of RTVP-1 in hNSCs is sufficient to trigger mesenchymal phenotypes. Transduction of hNSCs with a lentivirus vector expressing RTVP-1 markedly inhibited their neural differentiation when plated on poly-L-lysine coated plates in the absence of EGF and FGF, and maintained these cells as undifferentiated neurospheres (Fig. 5A). Furthermore, overexpression of RTVP-1 induced downregulation of the neuronal *marker β3-tubulin* and the astrocyte marker GFAP and induced the expression of the mesenchymal markers CD44, CTGF, FN and α-SMA (Fig. 5B). Moreover, overexpression of RTVP-1 resulted in the mesenchymal differentiation of these cells to adipocytes and osteoblasts as indicated by the increased presence of oil red-O and alizarin red S-positive cells (Fig. 5C). RTVP-1 also significantly increased hNSC cell migration as determined by transwell migration assay (Fig. 5D). Collectively, these findings demonstrate that overexpression of RTVP-1 in hNSCs inhibited neural differentiation and promoted mesenchymal differentiation of these cells.

### Glioma global gene network changes induced by silencing of RTVP-1

**Figure 6:**
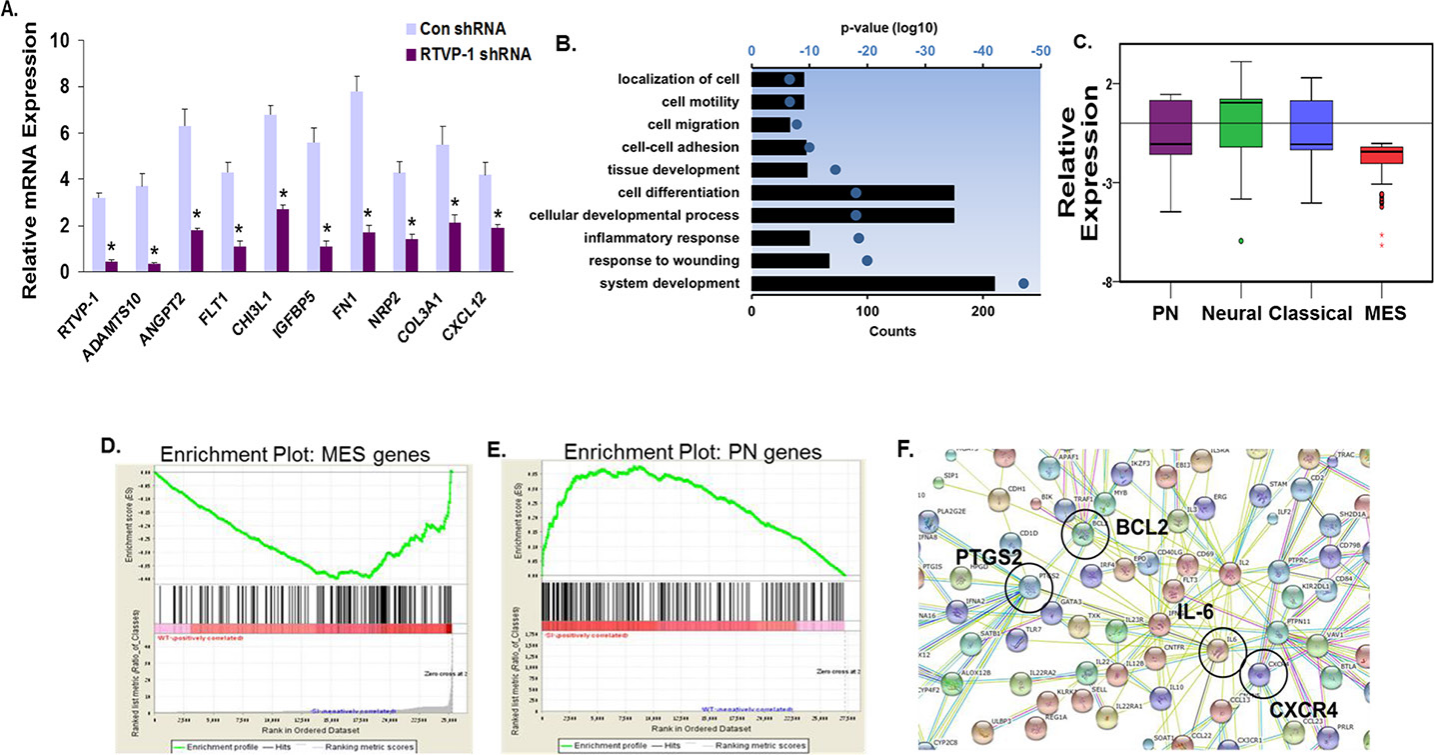
Global gene network changes induced by silencing of RTVP-1 in glioma cells U87 cells were silenced for RTVP-1 and after 3 days gene array analysis was performed as described in the methods. The expression of RTVP-1 and additional 8 genes that were downregulated in RTVP-1 silenced cells was validated using qRT-PCR **A.** *P < 0.001. Bar graph showing the top 10 gene ontology (GO) terms associated with the three-fold or greater of RTVP-1-silenced genes in U87 cells is presented as ranked by *P*-values. The axis represents the log10 of the *P*-value as determined by DAVID functional analysis **B.** The box plot represents the average expression levels of all the genes in the different glioma subtypes (i.e., Verhaak metagene scores for proneural, neural, classical and mesenchymal gene lists) in U87 glioma cells silenced for RTVP-1 **C.** GSEA analysis showing downregulation of genes associated with the mesenchymal phenotype **D.** and upregulation of genes associated with the proneural phenotype **E.** in RTVP-1-silenced U87 cells, with enrichment scores of −1.58 and 1.18, respectively; *P* (family-wise error rate) = 0.000; q (false discovery rate, FDR) = 0.000. A list of <3 fold and >3 fold of altered genes in RTVP-1 silenced cells was submitted to the STRING database. A functional protein association network was created using the parameters custom confidence level 0.24 and showing no more than five integrators. The type of protein–protein association (neighborhood, gene fusion, concurrence, experimental data, databases, text mining) corresponds to the connection color **F.**

To delineate the mechanisms by which RTVP-1 induces and maintains the mesenchymal transformation of glioma cells, we subjected U87 glioma cells silenced for RTVP-1 to microarray analyses. We found 3498 genes that were significantly downregulated (<3 fold) and 142 genes that were significantly upregulated (>3 fold) in U87 cells silenced for RTVP-1 compared to cells expressing a control shRNA. qRT–PCR analysis confirmed the downregulation of several microarray inferred mesenchymal genes in the RTVP-1 silenced cells (Fig. 6A). Further analyses of functional pathways using DAVID revealed that silencing of RTVP-1 induced downregulation of functional groups related to mesenchymal phenotype and to cell locomotion (Fig. 6B). To further confirm the pivotal role of RTVP-1 in regulating the mesenchymal phenotype of glioma cells we calculated the average expression levels of all the genes in the different subtypes of glioma (according to Verhaak proneural, neural, classical and mesenchymal gene lists [10]) and found that silencing of RTVP-1 induced a significant (*P* < 0.0001) downregulation in the expression of the mesenchymal genes compared to genes associated with proneural, neural and classical glioma subtypes (Fig. 6C).

We next used gene set enrichment analysis (GSEA) to identify gene sets that were enriched in the RTVP-1 silenced U87 cells. This analysis further confirmed downregulation of mesenchymal gene expression (Fig. 6D) and upregulation of proneural gene expression (Fig. 6E). In addition, GSEA revealed that RTVP-1 silencing in glioma cells induced downregulation of several pathways including those associated with stemness, such as IL-6, CXCR4 and NKT (Suppl. Table S2).

We next analyzed the downregulated (<3 fold) and the upregulated (>3 fold) gene using STRING (http://string-db.org/) and found four main gene networks consisting of IL-6, BCL2, PTGS2/COX-2 (prostaglandin endoperoxide synthase 2 gene) and CXCR4, that were regulated in glioma cells silenced for RTVP-1 (Fig. 6F).

### RTVP-1 mediates the mesenchymal transformation of glioma cells via the IL-6 pathway

Based on our GSEA and STRING analyses, we first focused on the role of IL-6 in the effects of RTVP-1 on the mesenchymal transformation of glioma cells. The IL-6 pathway is aberrant in GBM, correlated with the degree of malignancy and supports cell proliferation, migration, tumor growth and GSC tumorigenesis [33–35].

**Figure 7:**
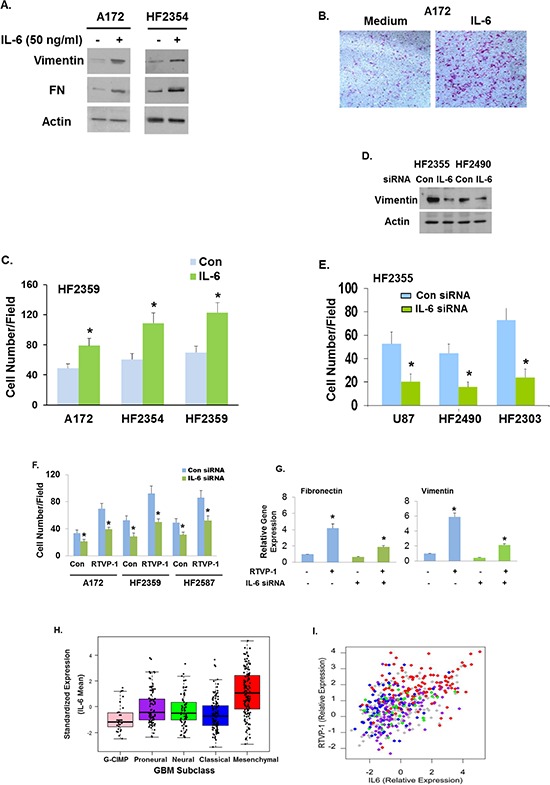
RTVP-1 mediates the mesenchymal transformation of glioma cells via the IL-6 pathway A172 glioma cells and the HF2354 GSCs were treated with 50 ng/ml of IL-6 for 48 h. The expression of vimentin and fibronectin was analyzed using Western blot analysis **A.** A172 cells **B.** and the HF2354 and HF2359 GSCs **C.** were analyzed for cell migration using transwell migration assay. U87 and GSCs were silenced for IL-6 or transfected with a control siRNA duplex. The expression of vimentin was analyzed using Western blot analysis **D.** and cell migration was analyzed using transwell migration assay **E.** The migration of A172 cells and GSCs silenced for IL-6 and overexpressing RTVP-1 was analyzed using transwell migration assay **F.** Mesenchymal marker expression was analyzed for the HF2587 GSCs as determined by RT-PCR **G.** IL-6 gene expression in the different subtypes of GBM was analyzed in TCGA dataset of 418 GBM patients **H.** Scatter plot of RTVP-1 expression versus IL-6 expression was generated using the TCGA dataset **I.** Each point is colored by the respective Verhaak classification with mesenchymal (red), classical (blue), neural (green) and proneural (purple). Tumors with unknown classification are grey. The results are representative of three different experiments that gave similar results. *P < 0.001.

We first examined whether IL-6 can induce mesenchymal transformation of glioma cells. For this analysis we treated A172 glioma cells and the HF2354 GSCs with IL-6 and found that this treatment upregulated the expression of the mesenchymal markers vimentin and FN in these cells (Fig. 7A), increased cell migration of the A172 cells and of the GSCs, HF2354 and HF2359 (Fig. 7C). Moreover, silencing of IL-6 downregulated the expression of the mesenchymal marker vimentin in the HF2355 and HF2490 GSCs (Fig. 7D) and inhibited cell migration of U87 glioma cells and of the HF2490 and HF2303 GSCs (Fig. 7E).

We next examined whether the mesenchymal transformation of glioma induced by RTVP-1 was mediated by IL-6. For this experiment we silenced the expression of IL-6 in A172 cells and examined the effects of RTVP-1 overexpression in the silenced cells. Silencing of IL-6 abrogated both cell migration (Fig. 7F) and the expression of mesenchymal markers, FN and vimentin induced by overexpression of RTVP-1 (Fig. 7G).

Using the TCGA data portal we analyzed the relative expression of IL-6 in the different subtypes of GBM patients. We found that similar to RTVP-1, mean expression levels of IL-6 were significantly higher in mesenchymal GBM (*P* < 0.0001) compared to the GCIMP, proneural, neural or classical subtypes (Fig. 7H). The expression of IL-6 in the GCIMP GBM was significantly lower compared to all other subtypes (Table S3). Moreover, the expression of RTVP-1 was positively correlated with the expression to IL-6 in GBM specimens (Pearson correlation: 0.512, *P* < 0.0001) and the relative expression of both appeared to be higher in the mesenchymal subtype (Fig. 7I).

In summary, these data strongly demonstrate that the mesenchymal transformation of glioma cells induced by RTVP-1 is, at least partly, mediated by the IL-6 pathway.

### RTVP-1 silencing in GSCs prolongs the survival of mice bearing intracranial xenografts

**Figure 8:**
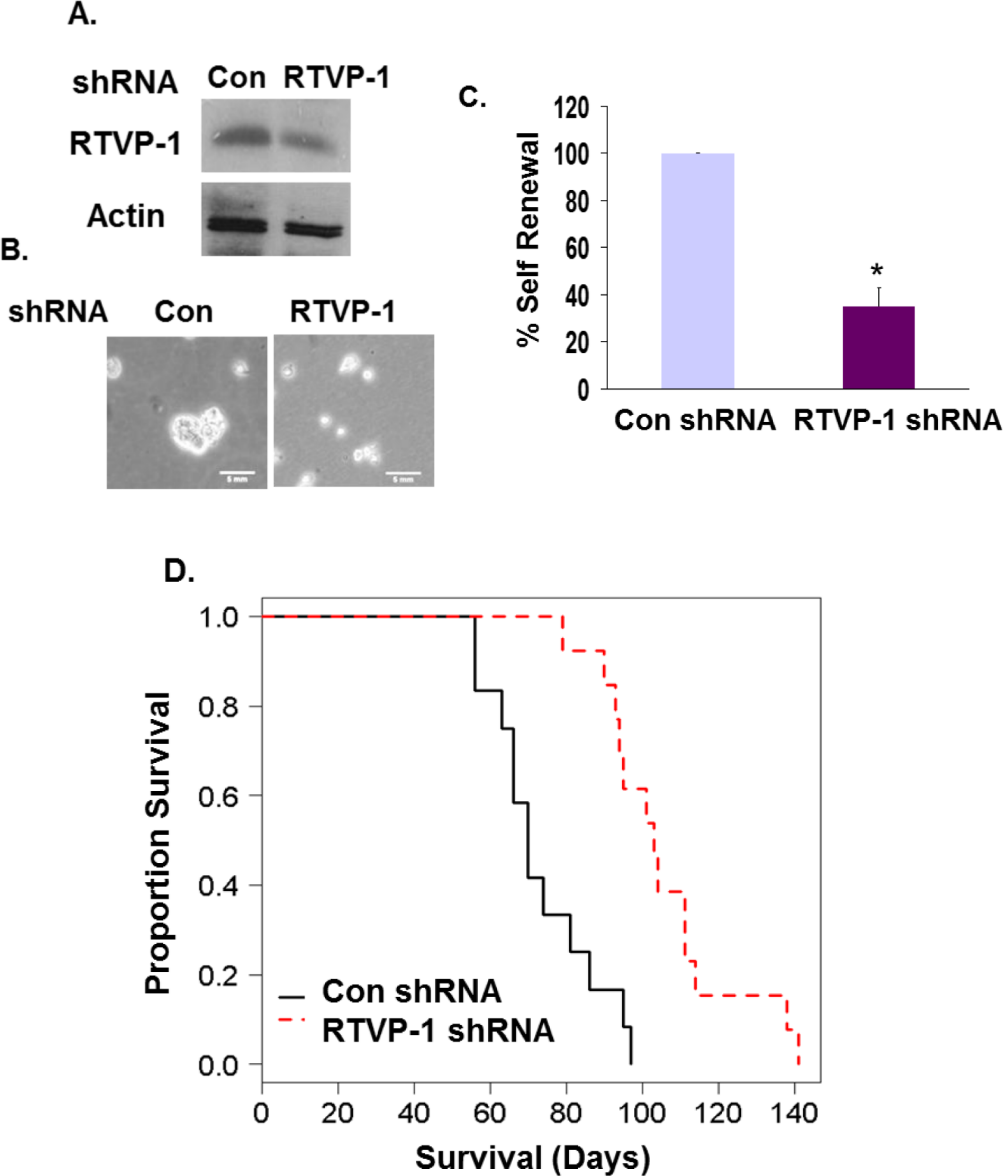
Silencing of RTVP-1 in GSCs prolongs the survival of mice bearing GSC-derived intracranial xenografts HF2303 GSCs were silenced for RTVP-1 using lentivirus vectors expressing specific shRNAs. RTVP-1 expression was determined using Western blot analysis **A.** Cell morphology is demonstrated using phase contrast microscopy **B.** and neurosphere formation analysis was determined after 14 days **C.** *P < 0.001. Kaplan-Meier survival curves of mice bearing intracranial xenografts derived from the HF2303 GSCs silenced for RTVP-1 or control shRNAs (*N* = 12) was determined **D.** *P* < 0.001.

Our results demonstrate an important role of RTVP-1 in the mesenchymal transformation and stemness of GSCs. We therefore examined the impact of RTVP-1 silencing on the tumorigenic capacity of GSCs *in vivo*. For these experiments we employed the GSC HF2303 that exhibits strong and stable mesenchymal characteristics. Since complete silencing of RTVP-1 in GSCs arrested cell growth and neurosphere formation (data not shown), we employed GSCs in which RTVP-1 was silenced by about 60% (Fig. 8A). These cells exhibited decreased neurosphere formation as described previously for other GSCs (Fig. 8B, 8C). Intracranial implantation of these cells into immunocompromised mice significantly increased the mean survival of tumor-bearing mice compared to the control group (107.8 days vs. 73.3 days, *P* = 0.00005) (Fig. 8D). These data demonstrate that RTVP-1 plays a major role in promoting the tumorigenic capacity of GSCs *in vivo* similar to its role in tumor aggressiveness in GBM patients (Fig. 1D).

## DISCUSSION

Despite recent advances in therapeutic approaches for the treatment of GBM, the survival of patients diagnosed with these tumors remains dismal. This is mainly attributed to the high level of infiltration and treatment resistance of residual GSCs, which eventually lead to tumor recurrence. GBM have been classified into several subtypes based on gene expression profile, with the two subtypes, proneural and mesenchymal, being the most robust and generally consistent among the classification schemes [9, 10, 15, 36].

Recent studies have shown that during glioma progression and recurrence, tumor cells acquire mesenchymal and lose proneural gene expression signature. The mesenchymal transformation of GBM is associated with tumor aggressiveness, therapy resistance and poor clinical outcome [9, 13, 14]. Thus, deciphering the mechanisms underlying the mesenchymal transformation of GBM is essential for identifying therapeutic targets and improving patient survival.

We previously reported that GLIPR1/RTVP-1 acts as tumor promoter in GBM and that its expression is increased in astrocytic tumors, in a grade-dependent manner [21]. Moreover, RTVP-1 plays a major role in promoting glioma cell migration and invasion. In this study we further characterized RTVP-1 expression and function in GBM, glioma cells and GSCs focusing on its role in the mesenchymal transformation of these cells.

Using gene expression data from the REMBRANDT [28] and TCGA data portals [27], we found that high expression of RTVP-1 in GBM specimens predicted poor prognosis. In addition, the expression of RTVP-1 was significantly higher in the mesenchymal GBM as compared to the other GBM subtypes. We next found that the mean expression of all genes associated with the mesenchymal signature was highly correlated with the expression of RTVP-1 in 259 GBM specimens, whereas the expression of genes that are associated with the proneural signature was inversely correlated with RTVP-1 expression. These data suggest that RTVP-1 may have a role in mediating the mesenchymal transformation of GBM.

Epithelial-to-mesenchymal transition (EMT) is essential for the development of malignant carcinoma metastasis and drug resistance [37–39]. Recently, mesenchymal transformation has also been shown to occur in GBM [40–43] and has been reported to be regulated by STAT3 and C/EBPβ [16]. We found the RTVP-1 promoter binds both STAT3 and C/EBPβ and that these TFs regulated the RTVP-1 promoter activity and RTVP-1 expression. Moreover, treatment of glioma cells with IL-6 upregulated RTVP-1 expression and activation of the RTVP-1 promoter in glioma cells via activation of STAT3. Using TCGA data analysis, we found a positive correlation between the expressions of STAT3, C/EBPβand RTVP-1 in GBM specimens, supporting a role of these TFs in the regulation of RTVP-1 expression. While the function of C/EBPβ in glioma progression is less defined, STAT3 has a well-characterized role in cancer progression and its activation in GBM is associated with the aggressive mesenchymal subtype and poor overall survival [33, 44, 45]. Targeting C/EBPβ and STAT3 in GBM patients may be challenging since these TFs regulate important functions in the normal brain. STAT3 was found to induce astrocytic differentiation and to inhibit neuronal differentiation of neural stem/progenitor cells, and C/EBPβ was found to promote neurogenesis and inhibits oligogenesis [46–49]. Therefore, the identification of tumor-specific pathways downstream of these TFs may have important mechanistic and therapeutic implications.

Our findings that RTVP-1 was preferentially expressed in the mesenchymal subtype of GBM and predicted poor clinical outcome and the fact the promoter of RTVP-1 contains binding sites for STAT3 and C/EBPβ led us to examine the involvement of this protein in the regulation of the mesenchymal phenotype of GBM. We found that overexpression of RTVP-1 induced a mesenchymal phenotype and differentiation in glioma cells, whereas silencing of RTVP-1 inhibited the mesenchymal signature of these cells, suggesting a role of RTVP-1 in the mesenchymal transformation process.

GBM contain a small subpopulation of self-renewing and highly tumorigenic cancer stem cells (GSCs) which contribute to therapy resistance and tumor recurrence and have been shown to be critical therapeutic targets [50–52]. More recently, it has been shown that GSCs can undergo a mesenchymal transformation in a TNF-α/NF-kB-dependent manner which confers resistance to radiotherapy [13]. We found that RTVP-1 is preferentially expressed in GSCs compared to normal hNSCs. Overexpression of RTVP-1 inhibited the neural differentiation and induced a significant mesenchymal transformation of GSCs, whereas, silencing of endogenous RTVP-1 in GSCs induced the opposite effects. Altogether, our results indicate that RTVP-1 mediated the mesenchymal transformation of GSCs and silencing of RTVP-1 inhibited the proneural-to-mesenchymal transit of these cells. Importantly, overexpression of RTVP-1 in normal hNSCs resulted in inhibition of the neural differentiation capacity and induced an acquisition of mesenchymal characteristics, indicating that RTVP-1 reprograms human NSCs along the mesenchymal lineage.

One of the main factors contributing to therapy failure in GBM is the activation of specific signaling pathways in GSCs which are associated with resistance to both chemo- and radiotherapy [13, 53, 54]. It was recently suggested that during therapy, GSCs can stay dormant and their high self-renewal capacity can cause local recurrence or an infiltrative lesion at a later time. It has also been suggested that stemness of tumor cells may be closely associated with mesenchymal transformation of these cells [30, 55–57]. Indeed, we recently demonstrated that RTVP-1 regulates the stemness of GSCs as determined by increased self-renewal and expression of the stemness genes Oct4 and Nanog and decreased differentiation ability downstream of miR-137 and upstream of the CXCR4-Shh-Gli pathway [29].

To examine the mechanism by which RTVP-1 promoted and maintained the mesenchymal transformation of glioma, we performed microarray analysis of gene expression of glioma cells silenced for RTVP-1. By combining analysis of microarray data and the average expression levels of all the genes of the different GBM subtypes in TCGA, we further confirmed that silencing of RTVP-1 inhibited the mesenchymal and induced the proneural phenotypes of glioma cells. Using GSEA we found that silencing of RTVP-1 induced downregulation of genes that are targets of the TFs STAT3 and C/EBPβ, which further supports our data that RTVP-1 is a downstream mediator of STAT3 and C/EBPβ effects on the mesenchymal transformation of glioma cells. Further analysis using STRING identified four main gene networks (IL-6, BCL2, PTGS2 and CXCR4) that were downregulated in glioma cells silenced for RTVP-1. The identification of CXCR4 as a major signaling molecule in these cells further confirmed our recent findings that miR-137 inhibited GSC self-renewal and promoted their differentiation by targeting RTVP-1 which downregulated CXCR4 [29].

IL-6 is a physiological activator of C/EBPβ and STAT3 that was found by us to upregulate RTVP-1 expression in a STAT3-dependent manner. We therefore focused on the role of the IL-6 pathway in RTVP-1 effects in glioma cells. The IL-6 pathway is known to be aberrant in GBM and to promote cell proliferation, migration and tumor growth *in vivo* [33, 58]. In addition, aberrant activation of STAT3 was associated with the tumorigenic effects of IL-6 in GSCs [34]. We found that similarly to RTVP-1, the expression of IL-6 correlated with the degree of malignancy of gliomas and that high IL-6 expression in GBM predicted poor prognosis [35].

Recently, IL-6 was shown to promote head and neck tumor metastasis by inducing EMT via the JAK-STAT3-SNAIL signaling pathway [59]. We found that silencing of IL-6 reduced the mesenchymal phenotype of glioma cells and GSCs, whereas treatment of the cells with IL-6 induced an opposite effect. More interestingly, silencing of IL-6 abrogated the effects of RTVP-1 on glioma cell migration and on the expression of the mesenchymal markers fibronectin and α-SMA. The existence of this IL-6-RTVP-1 interaction and its role in the mesenchymal transformation of GBM was further supported by TCGA data analysis which demonstrated that similar to RTVP-1, IL-6 was also preferentially expressed in the mesenchymal GBM subtype. More interestingly, we found that the expression of RTVP-1 was highly correlated with that of IL-6 in GBM specimens particularly in the mesenchymal subtype. In summary, these results strongly suggest that RTVP-1 mediates the mesenchymal transformation of glioma cells, at least partly, via the IL-6 pathway and points to the existence of positive regulatory pathways linking RTVP-1 and IL-6 via the activation of the STAT3 pathway.

GBM patients exhibit poor prognosis due to therapy resistance and invasiveness of residual tumor cells and tumor recurrence after resection, characteristics that are attributed to GSCs. Recently, it was reported that neural differentiation of GSCs decreased their tumorigenic potential and rendered them more susceptible to different therapies [60]. Thus, silencing of RTVP-1 in GSCs which decreases their self-renewal and promotes the neural differentiation of these cells, can be employed as a novel therapeutic approach to abolish the oncogenic potential of GSCs with no damage to normal cells in the tumor microenvironment [50].

We further examined the role of RTVP-1 in the tumorigenic potential of GSCs. Since complete knockdown of RTVP-1 in GSCs significantly inhibited their cell growth and neurosphere formation, we employed stable clones of GSCs that were only partially silenced for RTVP-1 expression. We found that partial knockdown of RTVP-1 expression in primary GSCs significantly increased the mean survival of GSC-derived xenograft-bearing mice. This finding suggests that in addition to the role of RTVP-1 in maintaining the mesenchymal phenotype and the stemness potential of GSCs, it also maintained the tumorigenic potential of GSCs *in vivo*. Analyzing data of GBM patients from the TCGA, a recently published study showed that long delay in tumor recurrence is associated with absence of the mesenchymal gene expression signature [14], raising the possibility that inhibiting this transformation may improve GBM patient prognosis. TCGA analysis demonstrated that patients with GBM expressing low levels of RTVP-1 have significantly prolonged disease-free survival (equal to prolonged time to tumor recurrence) compared to GBM patients expressing high levels of this gene. Moreover, lower expression of RTVP-1 in GBM appears to be a more significant predictive factor of prolonged disease-free survival than the absence of mesenchymal signature.

In summary, our results implicate RTVP-1 as an important prognostic marker and major effector in the mesenchymal transformation of GBM, glioma cells and GSCs downstream of STAT3 and C/EBPβ. This study also demonstrates an important positive feedback regulation of the IL-6-RTVP-1 pathway that promotes glioma cell migration, GSC stemness and the mesenchymal transformation of GBM.

## MATERIALS AND METHODS

### Cell cultures

The glioma cell lines A172, U251 and U87 were obtained from the American Type Culture Collection (Manassas, VA). Cells were maintained as previously described [29].

### Generation of primary GSC cultures

All human materials were used in accordance with the policies of the Institutional Review Board at Henry Ford Hospital. Generation of GSCs from fresh GBM specimens was performed as previously described [61]. The GSCs were examined for the expression of CD44, Bmi-1, CD133, Sox2 and nestin, self-renewal, expression of astrocytic, oligodendrocytic and neuronal markers upon differentiation and for their tumorigenic potential in nude mice [29, 40, 62–64].

### Neural and mesenchymal differentiation

For induction of neuronal differentiation, GSC neurospheres were seeded on poly-L-lysine (Sigma-Aldrich)-coated slide glasses and cultured for 10 days with DMEM containing 10% FCS as previously described [29]. Mesenchymal differentiation was assessed in glioma cell lines, GSCs and hNSCs. Adipogenic, osteogenic and chondrogenic differentiation was examined by incubating the cells with a StemPro^®^ Differentiation Kit (Invitrogen Life Technologies, USA) for 3 weeks. Adipogenic differentiation was analyzed by staining with oil red-O solution (Sigma-Aldrich), osteogenic differentiation and mineralization, by staining with alizarin red (Sigma-Aldrich) and alcian blue staining (Sigma-Aldrich) was used to assess chondrogenic differentiation.

### Immunofluorescence staining

For immunofluorescence staining, cells were fixed with 4% paraformaldehyde for 20 min and permeabilized with wash solution (0.1% Triton X-100 and 1% bovine serum albumin in PBS) for 20 min. Subsequently, cells were incubated with the specific first antibody for 1 h, washed three times with wash solution, and incubated with the subsequent secondary antibodies and with DAPI (Molecular Probes) for nuclear staining. Stained cells were observed using an eight-bit, 512 × 512 pixel, confocal Zeiss LSM510 microscope and AIM software.

### Transduction and transfection of GSCs, NSCs and glioma cell lines

Lentivirus vectors (System Biosciences, Mountain View, CA) expressing RTVP-1 or control, RTVP-1, STAT3 and C/EBPβ shRNAs were packaged and used to transduce the cells according to the manufacturer's protocol and as previously described [29]. In addition, GSCs, NSCs and glioma cell lines were transduced with adenovirus vectors expressing RTVP-1 or control vector at 5 multiplicity of infection (MOI) for 2 h. The medium was then replaced with fresh medium and the cells were used 48-h post infection. For each experiments, we employed two different shRNA constructs that gave similar results.

Cells were transfected either with the pcDNA3.1 control vector or with RTVP-1, C/EBPβ, STAT3 or IL-6 vectors (all plasmids were obtained from Addgene (Cambridge, MA) by electroporation using the Nucleofector device, protocol number A29 [Amaxa Biosystems, Gaithersburg, MD]).

### Small interfering RNA transfection

Small interfering RNA (siRNA) duplexes were synthesized and purified by Dharmacon (Lafayette, CO). The siRNA sequence for targeting RTVP-1 mRNA was 5′-AAGACTGCGTTCGAATCCATA-3′ (siRNA1). In addition, for some of the experiments we used pools of four siRNA duplexes for RTVP-1 and IL-6 and for the TF STAT3 and C/EBPβ that were also obtained from Dharmacon. Transfection of siRNAs was done using Oligofectamine (Invitrogen) according to the manufacturer's instructions. Experiments were performed 72 h after transfection.

### Western blot analysis

Cell pellet preparation and Western blot analysis were performed as previously described [22, 29].

### Scratch wound healing assay

Cells were plated in a 6-well plate and 48 h later at a confluence of 100% a migration gap of approximately 1 mm was created. The cells were washed with PBS and fresh media were added to remove any loose cells. Images of the initial scratch and at 16-h post-scratch were acquired using OLYMPUS IX50 microscope and 4x objective. To analyze cell migration into the scratched area, an average of 10 images was evaluated using the ImageJ software.

### Transwell migration assay

Transwell chambers (BD Biosciences, San Jose, CA) were used for analyzing cell migration as recently described [22].

### Luciferase reporter assay

Glioma cells were co-transfected with 3 μg of the firefly reporter construct and the desired expression plasmids or the Renilla control construct by electroporation using the Nucleofector device (Amaxa Biosystems, Germany). Firefly luciferase activities of the transfected cells were determined 24 h after the transfection using the dual luciferase assay system (Promega, Madison, WI) according to the manufacturer's instructions and as previously described [22].

### Chromatin immunoprecipitation assay (ChIP)

Chromatin immunoprecipitation assay (ChIP) was performed using a chromatin immunoprecipitation assay kit (Upstate, MA) following the manufacturer's instructions and as previously described [22]. Prior to the IP procedure, 10% of the supernatant was saved as total input chromatin and was processed with the eluted IPs beginning with the cross-linking reversal step. After the final ethanol precipitation, each IP or input sample was resuspended in 50 μl of PCR grade water.

### Real-time PCR

Total RNA was extracted using RNeasy midi kit according to the manufacturer's instructions (Qiagen). Reverse transcription reaction was carried out using 2 μg total RNA as described for the RT-PCR analysis. A primer optimization step was tested for each set of primers to determine the optimal primer concentrations. Primers, 25 μL of 2x SYBR Green Master Mix (Invitrogen), and 30 to 100 ng cDNA samples were resuspended in a total volume of 50 μL PCR amplification solution. The following primers were used:

BMP-2-forward GCGGAATTCGACTGCGGTCTCCTAAAGGTC, reverse GCGGCGGCCGCTTGCTGTACTAGCGACACCCAC; osteocalcin-forward ACACTCCTCGCCCTATTG, reverse GATGTGGTCAGCCAACTC; RUNX-2-forward CCAGAATGATGGTGTTGACG, reverse GGTTGCAAGATCATGACTAGGG; SOX9- forward CGAAATCAACGAGAAACTGGAC, reverse ATTTAGCACACTGATCACACG; BMP- 6-forward CAACAGAGTCGTAATCA, reverse TTAGTGGCATCCACAAGCTCT; FN- forward TGGCCAGTCCTACAACCAGT, reverse CGGGAATCTTCTCTGTCAGC; α-SMA-forward CCGACCGAATGCAGAAGGA, reverse ACAGAGTATTTGCGCTCCGAA; YKL-40 forward TGCCCTTGACCGCTCCTCTGTACC, reverse GAGCGTCACATCATTCCACTC; olig2-forward CAAATCTAATTCACATTCGGAAGGTTG, reverse GACGATGGGCGACTAGACACC CTGF-forward GGGAAATGCTGCGAGGAGT, reverse AGGTCTTGGAACAGGCGCTC; RTVP-1-forward TGCCAGTTTTCACATAATACAC, reverse GGATTTCGTCATACCAGTTT; CXCR4 - forward GTCATCTACACAGTCAACCTCTAC, reverse ACCACCTTTTCAGCCAACAG; Oct4 - forward ATCAGCCACATCGCCCAGCA, reverse CCCAGCAGCCTCAAAATCCT; Sox2-forward TGGGTTCGGTGGTCAAGTC, reverse CGCTCTGGTAGTGCTGGGA; S12-forward, TGCTGGAGGTGTAATGGACG, reverse CAAGCACACAAAGATGGGCT.

Reactions were run on an ABI Prism 7000 Sequence Detection System (Applied Biosystems, Foster City, CA). Cycle threshold (Ct) values were obtained from the ABI 7000 software. S12 or Δ-actin levels were also determined for each RNA sample as controls.

### TCGA data analysis

Level 2 processed gene expression data from the public-access clinical data tables were downloaded from the TCGA for 517 GBM. The per-sample files were merged on the Agilent probe ID to form a single data table (SAS v9.2). Expression values were assessed for each gene of interest (RTVP-1, C/EBPβ, STAT3, IL-6) and averaged across consistent probes. Expression was averaged within gene for persons who had more than one tumor sample/aliquot analyzed. Progression-free survival time is defined as the minimum time to progression or death with censoring at last follow-up time if neither had occurred at that time. For consistency, the origin of tissue was the brain, no prior tumor was recorded and the histopathology was noted to be untreated primary (de novo) GBM.

### REMBRANDT data analysis

All glioma patient data were publicly available in de-identified form and obtained from the NCI Repository for Molecular Brain Neoplasia Data (REMBRANDT) database (https://caintegrator.nci.nih.gov/rembrandt/), using the data available on April 22, 2013. There were 343 glioma patient samples that were correlated between RTVP-1 expression and overall survival. Although all of the patient samples are represented in the Kaplan–Meier Survival Plot, not all of the patients in the database were identified for glioma subtype. Thus, only those patients that were positively identified as astrocytoma or GBM were used in the analysis for calculating the percentage of patients with upregulated, intermediate or downregulated RTVP-1 expression. The differences between groups were analyzed by log-rank *P* value.

### Metagene calculations

The metagenes were determined from the 840 gene list of Verhaak [10]. The gene symbols were distributed between the 4 classes: Classical *N* = 162, Mesenchymal *N* = 216, Neural *N* = 129, and Proneural *N* = 178. Level 3 data files for the Agilent gene expression microarrays were acquired for all available GBM samples in the TCGA (July 2012, *n* = 593). For each tumor, the averaged expression of genes in each of the 4 class signatures was calculated generating 4 ‘metagene’ scores, one for each subtype for every tumor [65].

### Microarray analyses

RNA of U87 glioma cells transfected with a control of RTVP-1siRNA sequence was used in the hybridizations of Unrestricted AMADID Release GE 4x44K expression array chip according to the manufacturer's specifications (Agilent). Intensity data were obtained from array images and analyzed using R Suite.

### Bioinformatics analysis of the microarray results

Target genes that were identified to be associated with silencing of RTVP-1 in U-87 MG glioma cells were processed using the DAVID Web tool (http://david.abcc.ncifcrf.gov) to obtain association of these genes with specific GO functional categories. The resulting GO terms were ranked from smallest to largest *P*-values after removing GO terms that had 10 or fewer genes overlapping with the query gene list. For STRING analysis, the list of the gene targets was annotated and networks were generated using the STRING (Search Tool for the Retrieval of Interacting Genes) analysis tool. Each interaction in the database is annotated with a benchmarked numerical confidence score, which can be used to filter the interaction network at any desired stringency. GSEA was performed using the publicly available desktop application from the Broad Institute (http://www.broad.mit.edu/gsea/software/software_index.html). Genes represented by more than one probe were collapsed using the XCollapseProbes utility to the probe with the maximum value. The gene set database used was that of functional sets, s2.symbols.gmt. *P* values were calculated by permuting the genes 1000 times.

### GSC-derived xenografts

Following the guidelines of Henry Ford Hospital's IACUC, dissociated GBM neurospheres were inoculated intracranially in nude mice (Nu/Nu). Animals were anesthetized and a Hamilton syringe was used to inject the HF2303 GSCs transduced with lentivirus vector expressing RTVP-1 or control shRNAs through a 3-mm hole to the right of the bregma, at a depth of 2.5 mm, at a rate of 0.5 μL/30 s. The surgical zone was flushed with sterile saline, the hole sealed with bone wax, and the skin over the injection site sutured. Animals were monitored daily and sacrificed at the end of the experiment.

### Statistical analysis

The results are presented as the mean values ± SD. The data of patient specimens are presented graphically with median and interquartile range noted. Data were analyzed using ANOVA or a Student's *t*-test with correction for data sets with unequal variances. An age-adjusted *t*-test was taken from a linear model including age as a covariate. Correlation was assessed using Pearson's correlation coefficient and tested against a correlation of zero (no correlation). The squared Pearson coefficient, or coefficient of determination (R^2^) for a single predictor regression, is given on the scatter plots. Kaplan-Meier analysis was used to produce survival curves with differences tested between groups by the log-rank test. Data were analyzed on a log 2 scale as appropriate.

## SUPPLEMENTARY FIGURES AND TABLES


