# Supplementary material for: RTVP-1 promotes mesenchymal transformation of glioma via a STAT-3/IL-6-dependent positive feedback loop
Source: Oncotarget. 2015 Jul 18;6(26):22680–97. doi: 10.18632/oncotarget.4205 (PMC4673191; doi:10.18632/oncotarget.4205)
Supplement: Supplementary file 1 [file oncotarget-06-22680-s001.pdf]

SUPPLEMENTARY FIGURES AND TABLES

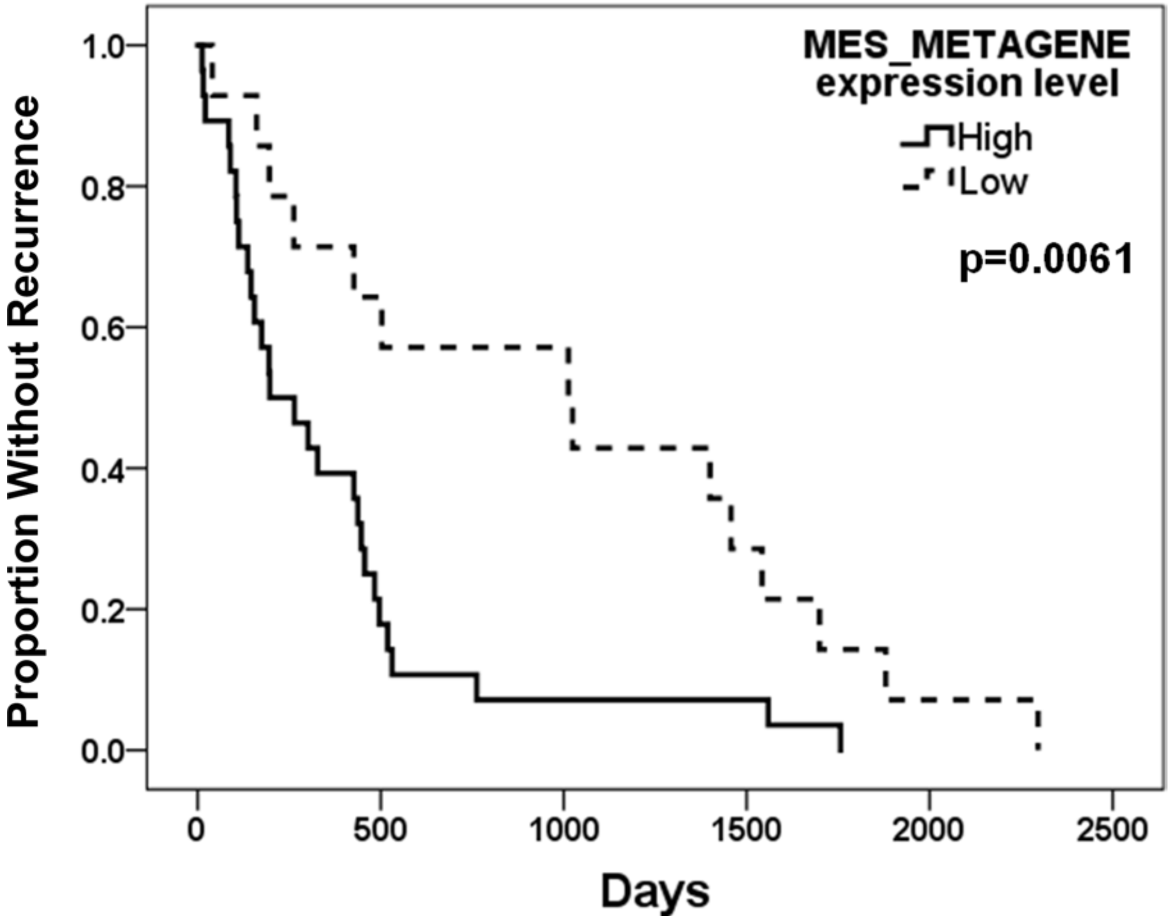

Supplementary Figure S1: Progression free survival as Kaplan-Meier curves for primary GBM specimens profiled by TCGA. The GBM specimens were grouped by the intensity of the mesenchymal metagene derived from the key genes defining the mesenchymal gene expression class. The two groups represent a split at the median intensity and were compared by log-rank test.

STAT3

-311
atttctttggaaatatctcccaatt caaaaggaagtctgacacagcctc
ataaaaatctatgtcgtaataggtgctgtgtcactgaaaaccactgaaa
agacagggttaagaacacaaagtgagctgcacaccatatatggagaaa
cccgtttcttaaaactagtgatgaactcatgctctgttctgttttctcaaa
gctgaagtcggctaggtttgcaaagctgtgggctgagcactcaggcaat
cacactctcagaaactgcggcggctctggactgcagcctcccaaggctc
catgccagacaaagc

CEBP/β

-311
atttctttggaaatatctcccaatt caaaaggaagtctgacacagcctc
ataaaaatctatgtcgtaataggtgctgtgtcactgaaaaccactgaaa
agacagggttaagaacacaaagtgagctgcacaccatatatggagaaa
cccgtttcttaaaactagtgatgaactcatgctctgttctgttttctcaaa
gctgaagtcggctaggtttgcaaagctgtgggctgagcactcaggcaat
cacactctcagaaactgcggcggctctggactgcagcctcccaaggctc
catgccagacaaagc

Supplementary Figure S2: Analysis of STAT3 and C/EBPβ in the RTVP-1 promoter. The RTVP-1 promoter was analyzed for transcriptional regulatory elements using the MatInspector software. Binding sites for STAT3 and C'/EBPβ are marked.

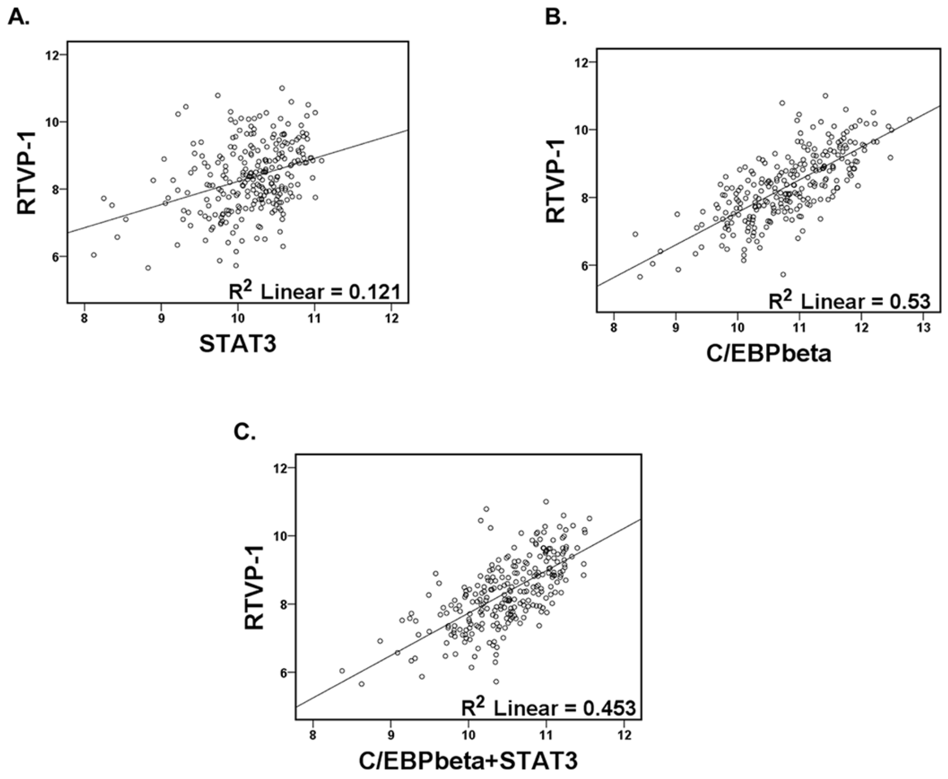

Supplementary Figure S3: Correlation of RTVP-1, STAT3 and C/EBPβ expression in GBM specimens. Scatter plots of RTVP-1 expression versus the expression of STAT3 (J), C/EBPβ (K) and C/EBPβ + STAT3 (L) were generated using TCGA dataset of 259 GBM specimens.

Supplementary Table S1. Expression of RTVP-1 in the different GBM subtypes

Subtype	*N*	Mean	SD	(Min, Max)
GCIMP	30	−0.02	1.26	(−2.02, 3.60)
Proneural	91	0.40	0.87	(−2.34, 2.20)
Neural	77	0.56	0.81	(−1.67, 2.52)
Classical	135	0.48	0.94	(−1.86, 2.94)
Mesenchymal	148	1.61	0.96	(−1.65, 3.63)

Distribution of RTVP-1 expression among the primary GBM cases profiled by TCGA. Data are given for the five gene expression subclasses of GBM as described by Verhaak et al. [10] and others [11].

Supplementary Table S2. Gene set enrichment analysis of U87 cells silenced for RTVP-1

	Gene sets enriched in MG-U87 targeted for RTVP-1	NES	Count
Manually Loaded	MSC GENES	157	−1.5895
	PN GENES	163	1.18
	IL-6 GENES	79	−1.5492
BioCarta Pathways	NKT PATHWAY	28	−1.5359
	STEM PATHWAY	15	−1.4853
	CYTOKINE PATHWAY	19	−1.4101
	INFLAM PATHWAY	28	−1.3508
	P53 HYPOXIA PATHWAY	19	−1.2872
	INTEGRIN PATHWAY	35	−1.2781
	PTEN PATHWAY	16	−1.1189
	IL6 PATHWAY	21	−1.0884
KEGG Pathways	HSA04140_ REGULATION_OF_ AUTOPHAGY	25	−1.6022
	HSA00590_ ARACHIDONIC_ACID_ METABOLISM	51	−1.5648
	HSA04610_ COMPLEMENT_AND_ COAGULATION_ CASCADES	68	−1.4464
	HSA04060_CYTOKINE_ CYTOKINE_ RECEPTOR_ INTERACTION	247	−1.4281
Motif gene sets(C3)/ transcription factor targets	V$RP58_01	174	−1.5387
	V$STAT3_01	16	−1.4527
	V$HNF1_01	56	−1.4464
	V$SMAD4_Q6	171	−1.4048
	V$SRF_01	47	−1.2907
	V$CEBPB_02	204	−1.1902

GESA of U87 cells silenced for RTVP-1 demonstrates downregulation of several pathways including those associated with stemness such as IL-6, CXCR4 and NKT.

Supplementary Table S3. Expression of IL-6 in the different GBM subtypes

Subtype	N	Mean	SD	(Min, Max)
GCIMP	30	−0.93	1.03	(−2.50, 1.45)
Proneural	91	−0.04	1.35	(−2.36, 3.77)
Neural	77	−0.29	1.23	(−2.43, 3.29)
Classical	135	−0.64	1.20	(−3.16, 3.63)
Mesenchymal	148	1.08	1.74	(−2.93, 5.09)

Distribution of IL-6 expression among the primary GBM cases profiled by TCGA. Data are given for the five gene expression subclasses of GBM as described by Verhaak et al. [10] and others [11].
